# Supplementary material for: A reconfigurable binary/ternary logic conversion-in-memory based on drain-aligned floating-gate heterojunction transistors
Source: Nat Commun. 2023 Jun 23;14:3757. doi: 10.1038/s41467-023-39394-5 (PMC10290076; doi:10.1038/s41467-023-39394-5)
Supplement: Supplementary file 1 — Supplementary Information [file 41467_2023_39394_MOESM1_ESM.pdf]

Supplementary Information

A reconfigurable binary/ternary logic conversion-in-memory based on drain-aligned floating-gate heterojunction transistors

Chungryeol Lee[1], Changhyeon Lee[1], Seungmin Lee[1], Junhwan Choi[2], Hocheon Yoo[*,3], and Sung Gap Im[*,1,4]

[1] *Department of Chemical and Biomolecular Engineering*
Korea Advanced Institute of Science and Technology (KAIST)
291 Daehak-ro, Yuseong-gu, 34141, Korea

[2] *Department of Chemical Engineering*
Dankook University
152, Jukjeon-ro, Suji-gu, Yongin, 16890, South Korea

[3] *Department of Electronic Engineering*
Gachon University
1342 Seongnam-daero, Seongnam, 13120, Korea

[4] *KAIST Institute for NanoCentury (KINC)*
Korea Advanced Institute of Science and Technology (KAIST)
291 Daehak-ro, Yuseong-gu, 34141, Korea

Correspondence and requests for materials should be addressed to H.Y. (hyoo@gachon.ac.kr) or S.G.I. (sgim@kaist.ac.kr).

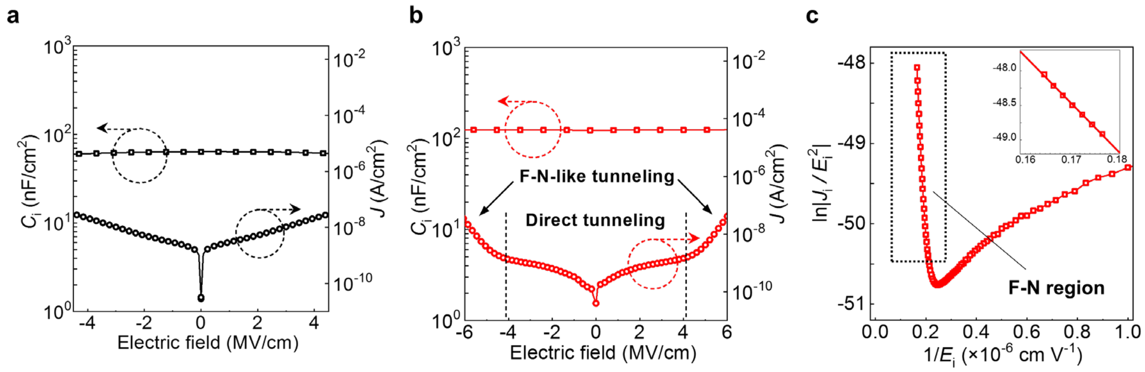

Supplementary Figure 1 | ** The insulating performance of the polymer dielectric layers; **a. Capacitance per unit area (C_i)–electric field (E) and leakage current density (J)–E characteristics of poly(2-cyanoethyl acrylate-*co*-diethylene glycol divinyl ether) [p(CEA-*co*-DEGDVE)] (pCD, ~70 nm) blocking dielectric layer (BDL) and **b**. poly(1,3,5-trivinyl-1,3,5-trimethyl cyclotrisiloxane) (pV3D3, ~15 nm) tunneling dielectric layer (TDL). **c**. ln|J_i/E_i^2|–1/E_i characteristics of pV3D3

For tunneling dielectric layer (TDL), we used poly(1,3,5-trivinyl-1,3,5-trimethyl cyclotrisiloxane) (pV3D3) which can be deposited by a vapor-phase polymer deposition process, termed initiated chemical vapor deposition (iCVD). The pV3D3 TDL showed the Fowler-Nordheim (F-N)-like tunneling behavior in the E range from ~4 MV/cm (Supplementary Fig. 1b, c), which is fully consistent with our previous report[29].

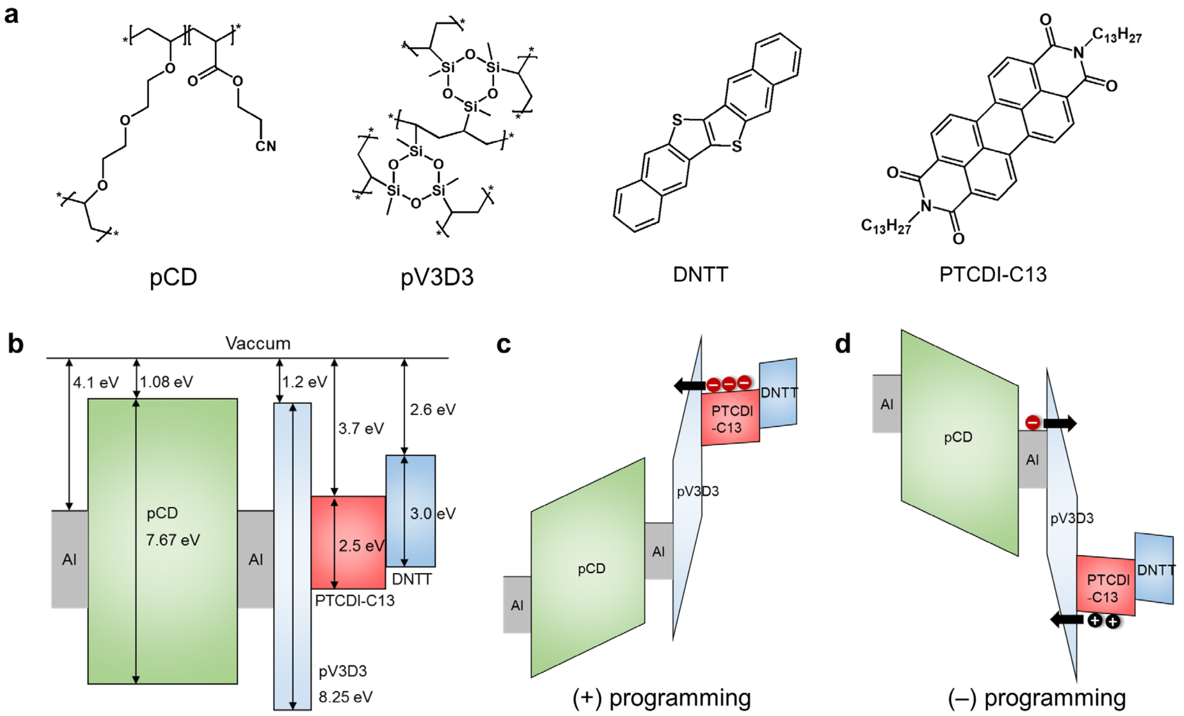

Supplementary Figure 2 | The materials and band structure of heterojunction non-volatile memory transistor (H-MTR); **a.** Chemical structures of the polymer dielectrics and organic semiconductors. Energy band diagram of H-MTR for **b**. Flat band, **c**. (+) programming, and **d**. (–) programming operations.

The lowest unoccupied molecular orbital (LUMO) level of *N,N'*-Ditridecyl-3,4,9,10-perylenetetracarboxylic diimide (PTCDI-C13) and pV3D3 TDL was 3.7 eV[17] and 1.2 eV[29], respectively, to form a 2.5 eV of barrier for electron. Also, 3.25 eV of barrier was generated for hole transfer between the highest occupied molecular orbital (HOMO) level of PTCDI-C13 (6.2 eV)[17] and that of pV3D3 TDL (9.45 eV)[29]. Such high energy barriers can be overcome by Fowler-Nordheim (F-N)-like tunneling under high *E*, thanks to the ultrathin (15 nm) pV3D3 TDL[30]. The sandwiched structure of the high bandgap dielectrics of pCD BDL (7.67 eV)[30] and pV3D3 TDL (8.25 eV) yielded a deep quantum well for Al floating-gate (FG) with the work function (WF) of 4.1 eV. Thus, energy band structure based on polymer dielectrics and organic semiconductors in Supplementary Fig. 2 ensured the reliable memory operation in that the charges can tunnel through the pV3D3 TDL according to the

applied programming voltage, while the trapped charges in the FG cannot tunnel through the pCD BDL and pV3D3 TDL during the transistor operation.

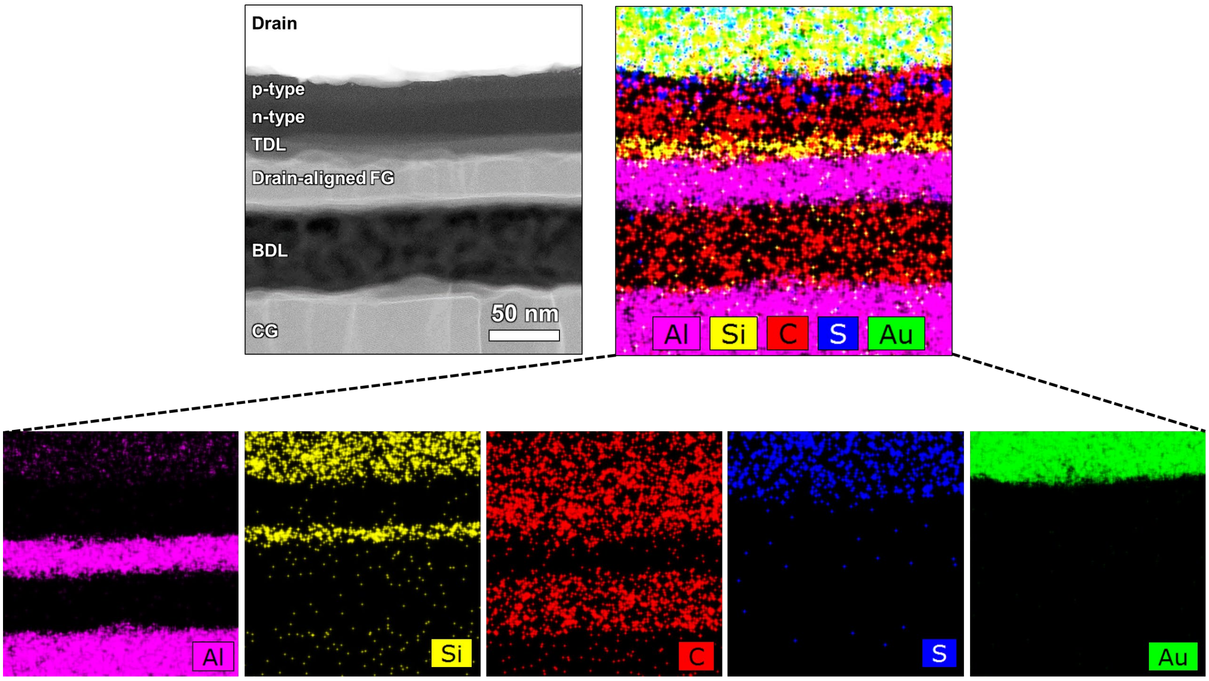

Supplementary Figure 3 | A high-resolution transmission electron microscope (HRTEM) and energy-dispersive spectroscopy (EDS) elemental mapping result of the heterojunction non-volatile memory transistor.

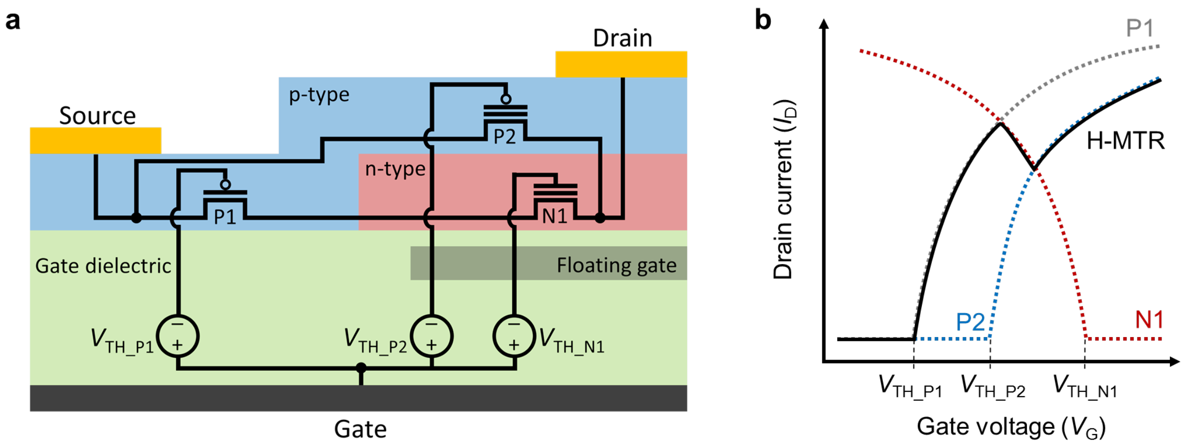

Supplementary Figure 4 | Schematic diagram illustrating charge carrier transports of the H-MTR; **a**. Equivalent circuit model and **b**. corresponding transfer characteristics of the H-MTR.

In H-MTR, there are two different charge carrier transport paths. Each path corresponds to charge flow through p-type and n-type lateral junction (Path 1) and charge flow through continuous p-type layer (Path 2). Here, the electrical characteristics of top p-type layer (P2) forming vertical heterojunction with n-type (N1) would be quite different from the bottom p-type (P1) in touch with dielectric layer. Therefore, equivalent circuit model of the H-MTR includes series connection of P1 and N1 transistors as well as P1 and P2 transistors, where both serially connected transistors (P1-N1 and P1-P2) are connected in parallel as well (Supplementary Fig. 4a). Considering the equivalent circuit, the ideal transfer characteristics of the H-MTR can be represented with three nominal threshold voltage; threshold voltage of P1 transistor (V_{TH_P1}), threshold voltage of P2 transistor (V_{TH_P2}) and threshold voltage of N1 transistor (V_{TH_N1}) (Supplementary Fig. 4b). From the transfer characteristics, V_{TH_P1} and V_{TH_P2} determine peak voltage (V_{Peak}) and peak current (I_{Peak}) while V_{TH_P1} and V_{TH_P2} govern valley voltage (V_{Valley}) and valley current (I_{Valley}) of the H-MTR. Since drain-aligned floating gate can adjust the V_{TH_P2} and V_{TH_N1} of the H-MTR according to its memory state, the NTC characteristics of V_{Peak}, V_{Valley}, I_{Peak}, I_{Valley} can be effectively controlled by programming operation.

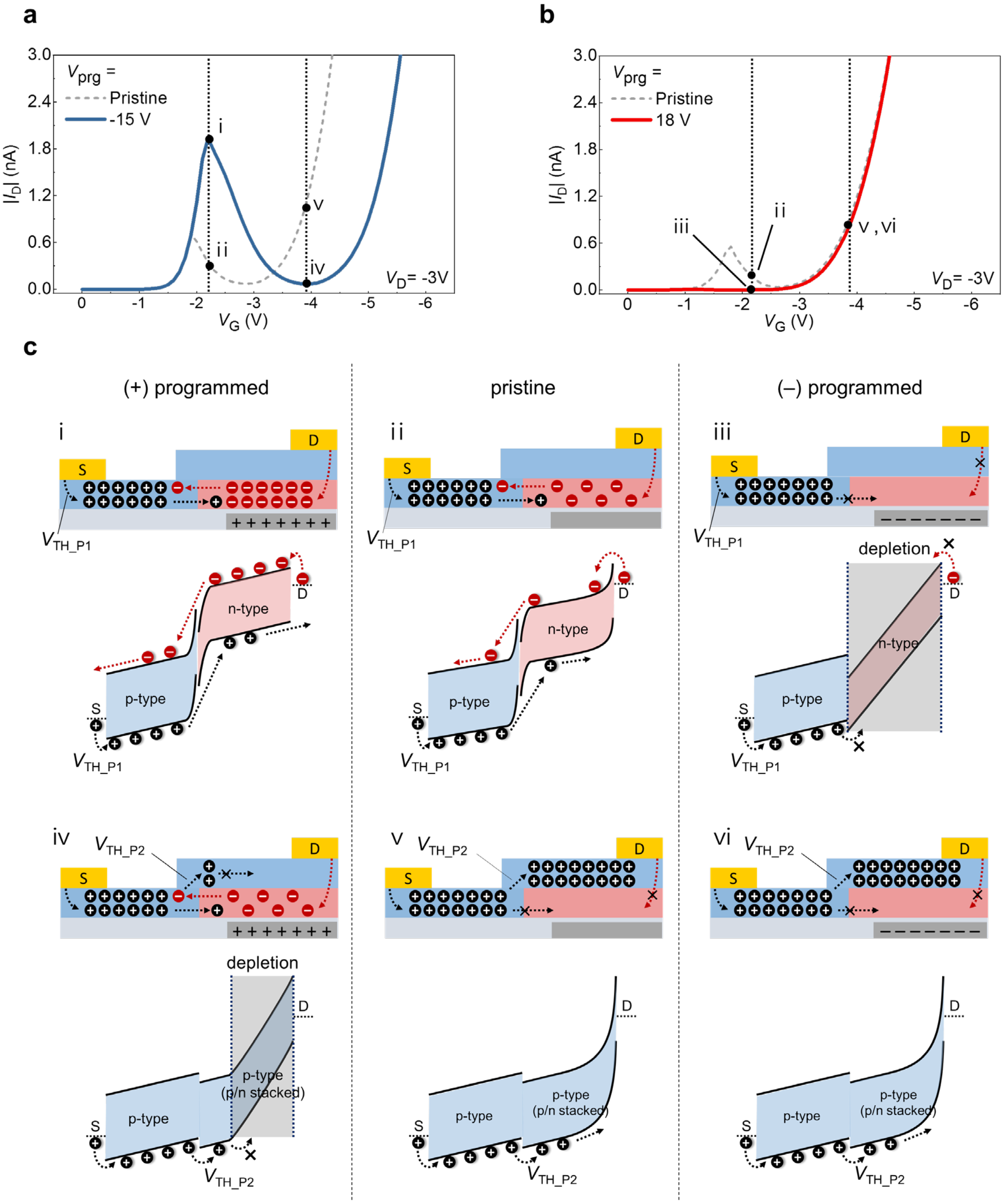

Supplementary Figure 5 | Schematic illustration and energy band diagram describing the charge carrier transports in H-MTR according to its memory state; **a**. The transfer characteristics of H-MTR with respect to programming voltage (V_{prg}). V_{prg} = –15 V and **b**. V_{prg} = +18 V. A dashed curve represents transfer characteristics of H-MTR in pristine state. **c**. Schematic illustrations and the energy-level alignments of pristine, (+) programmed, and (–) programmed H-MTR at specific gate voltage (V_G).

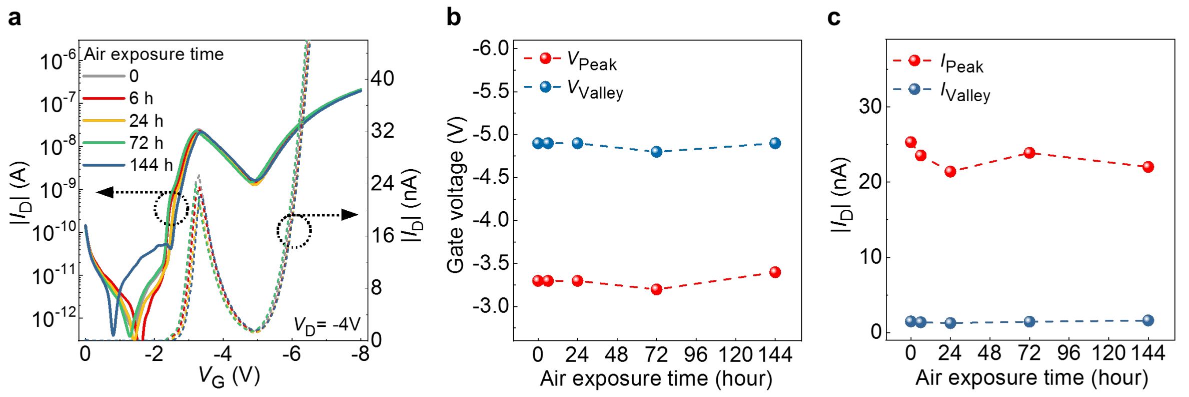

Supplementary Figure 6 | Air stability of the Al_2O_3 encapsulated H-MTR with respect to air exposure time; **a**. Transfer characteristics, **b**. V_{Peak} and V_{Valley}, and **c**. I_{Peak} and I_{Valley} of the H-MTR with respect to air exposure time.

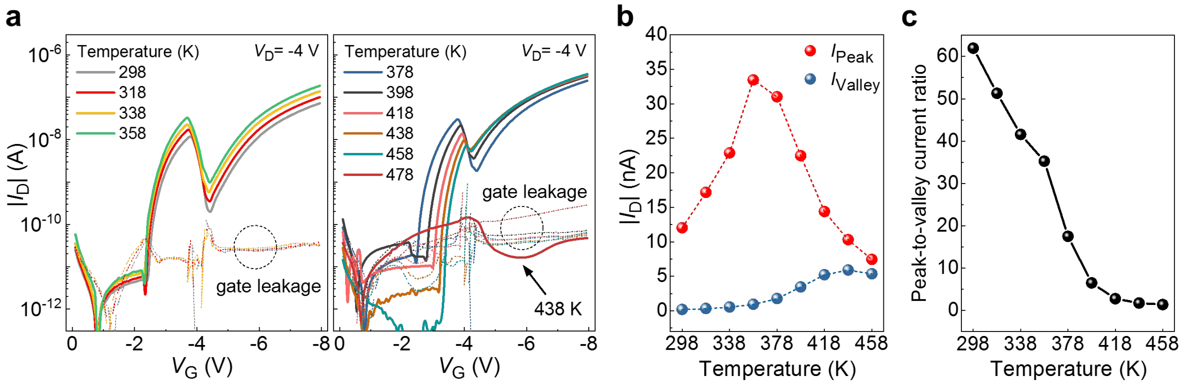

Supplementary Figure 7 | Temperature-dependent NTC characteristics of H-MTR; **a**. Drain current (I_D)–V_G plots, **b**. Extracted I_{Peak} and I_{Valley} and **c**. Extracted peak-to-valley current ratio of H-MTR as functions of temperature.

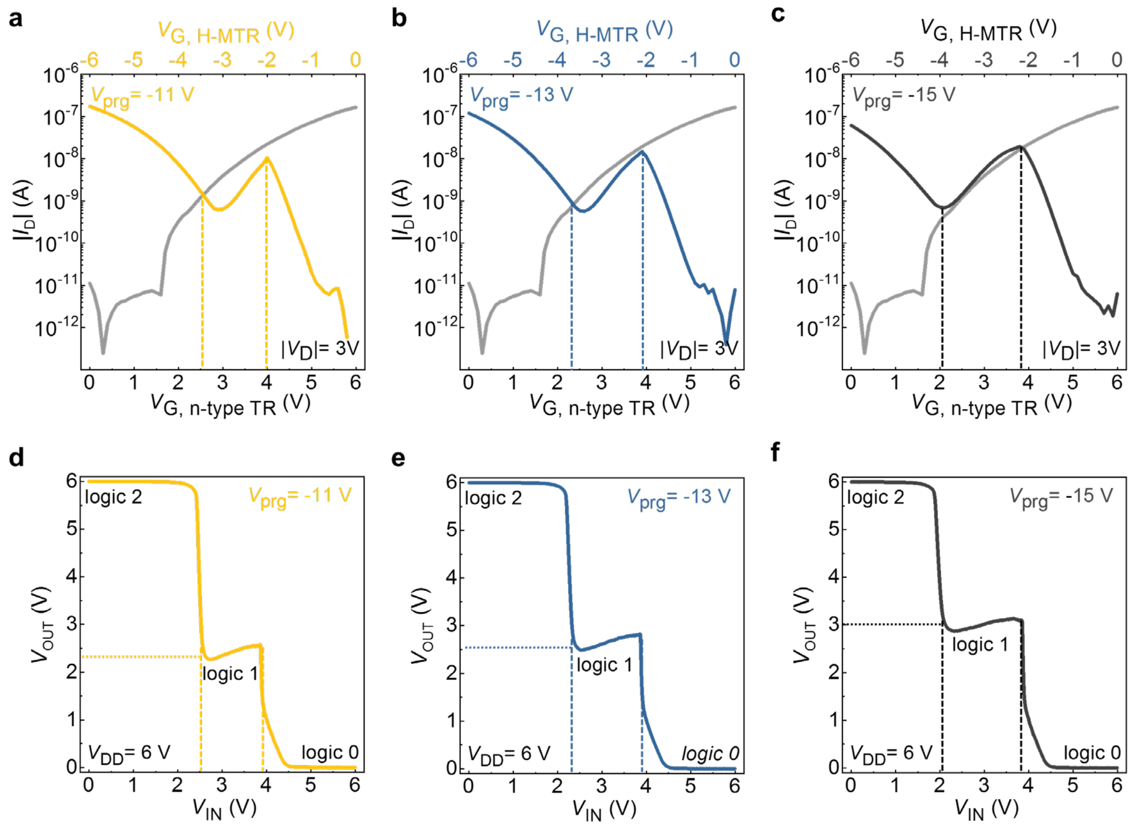

Supplementary Figure 8 | The overlapped transfer characteristics of the H-MTR and n-type transistor for ternary logic operation with respect to V_{prg}; **a**. V_{prg} = −11 V, **b**. V_{prg} = −13 V, and **c**. V_{prg} = −15 V.

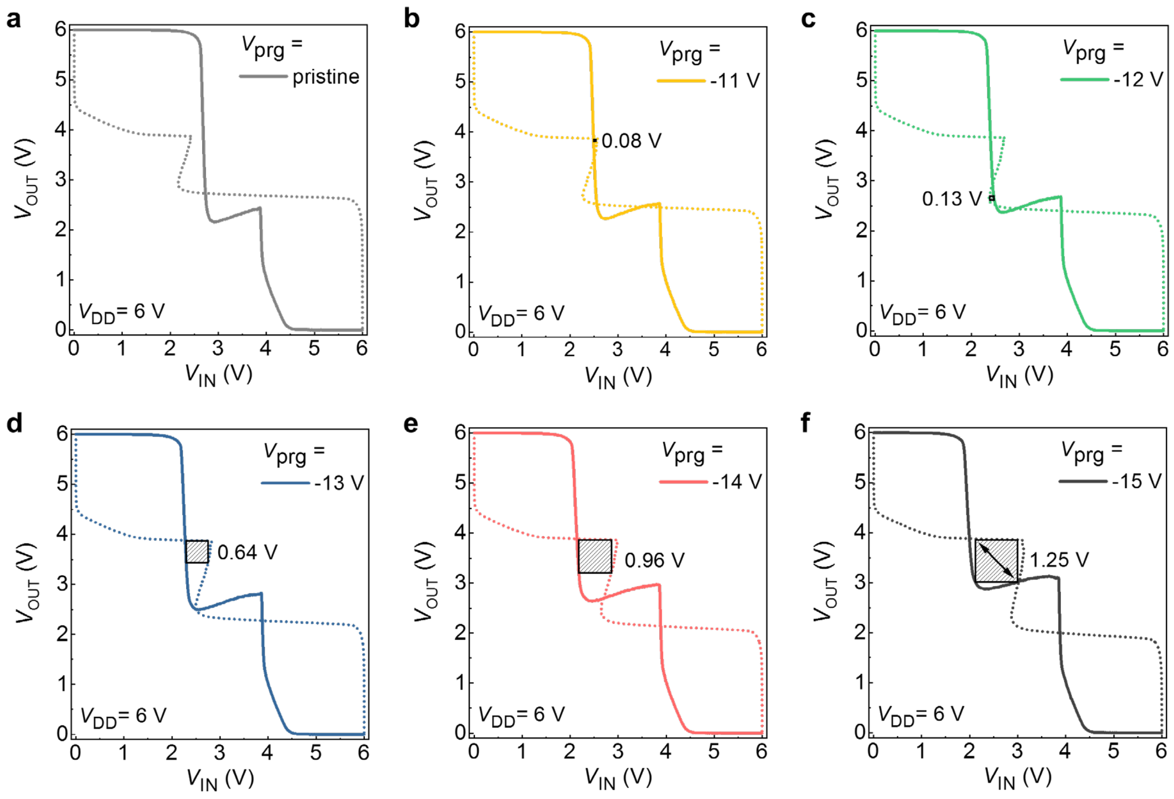

Supplementary Figure 9 | Static noise margin (SNM) of binary/ternary reconfigurable logic inverter (R-inverter) for ternary logic operation with respect to V_{prg}; **a.** initial state, **b.** V_{prg} = −11 V, **c.** V_{prg} = −12 V, **d.** V_{prg} = −13 V, **e.** V_{prg} = −14 V, and **f.** V_{prg} = −15 V.

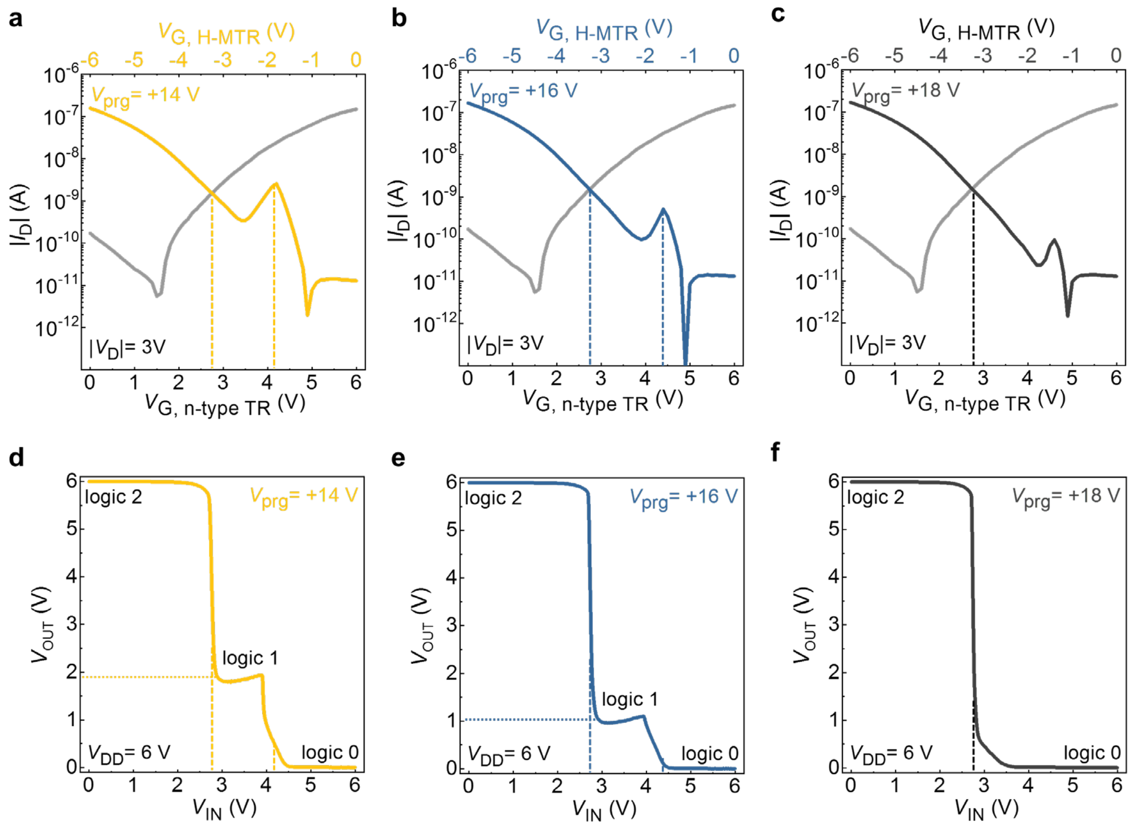

Supplementary Figure 10 | Overlapped transfer characteristics of the H-MTR and n-type transistor for ternary logic operation with respect to V_{prg}; **a**. V_{prg} = +14 V, **b**. V_{prg} = +16 V, and **c**. V_{prg} = +18 V.

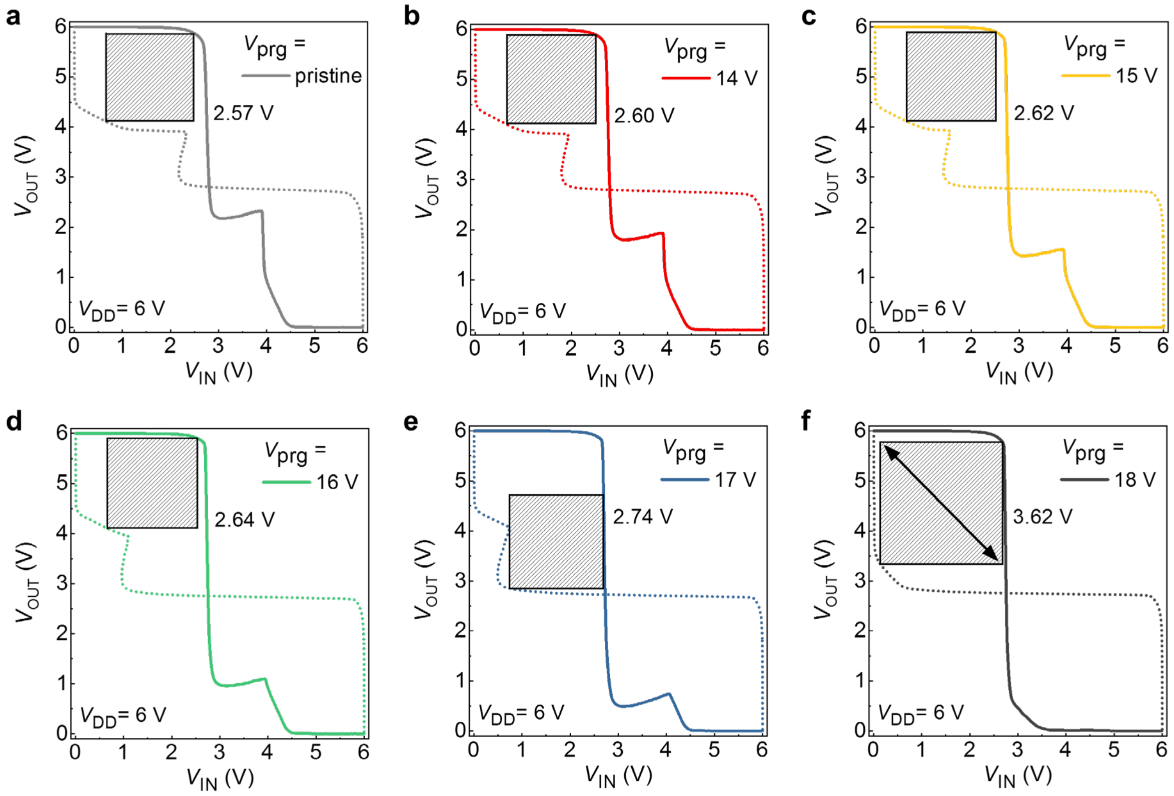

Supplementary Figure 11 | Static noise margin (SNM) of binary/ternary reconfigurable logic inverter (R-inverter) for binary logic operation with respect to V_{prg}; **a**. initial state, **b**. V_{prg} = +14 V, **c**. V_{prg} = +15 V, **d**. V_{prg} = +16 V, **e**. V_{prg} = +17 V, and **f**. V_{prg} = +18 V.

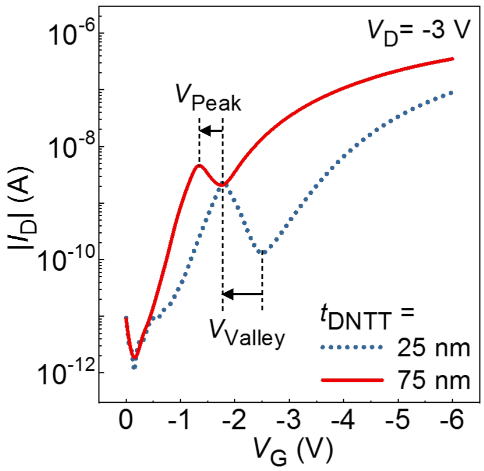

Supplementary Figure 12 | Transfer characteristics of H-MTR with respect to DNTT thickness (t_{DNTT}). t_{DNTT} =25 nm (dotted line) and 75 nm (solid line).

Since increasing the thickness of dinaphtho[2,3-b:2′,3′-f]thieno[3,2-b]thiophene (DNTT) layer (t_{DNTT}) can induce the higher conductance of p-channel, the t_{DNTT} was increased from 25 nm to 75 nm for H-MTR, and the transfer characteristic of the modified H-MTR with t_{DNTT} = 75 nm (solid line) shows that V_{TH_pp} and V_{TH_pn} were successfully shifted toward $+V_G$ direction compared to the original H-MTR with t_{DNTT}=25 nm (dotted line).

Supplementary Table 1 | Performance comparison with the reported multi-valued logic (MVL) devices with negative transconductance (NTC) characteristic.

H-TR Channel	Load Channel	V_{IN} [V]	V_{DD} [V]	Noise margin	MVL Application	Integration level	Ref
MoS_2/WSe_2	WSe_2	0 to 1	1	-	STI	-	[1]
MoS_2/BP	BP	−1 to 1	1	-	STI	-	[2]
WSe_2 /Graphene	WSe_2	−30 to 30	−4	-	STI	-	[3]
Graphene (R6G-doped)	Graphene	−40 to 40	1	-	STI	-	[4]
α-6T /PTCDI-C8	PTCDI-C8	−10 to 60	60	-	STI	-	[5]
SnSeS/BP	BP	−4 to 4	1	-	STI	-	[6]
$MoS_2/MoTe_2$	$MoTe_2$	−60 to 10	26	-	STI	-	[7]
$MoTe_2/MoS_2$	$MoTe_2$	−60 to 60	2	-	STI	-	[8]
$MoTe_2/MoS_2$	$MoTe_2$	−40 to 40	1	-	STI	-	[9]
PTCDI-C13 /DNTT	PTCDI-C13	0 to 50	50	-	STI	-	[10]
2H-$MoTe_2$ /BP	BP	−5 to 40	1	-	STI	-	[11]
InO/SWCNT	SWCNT FET	−0.5 to 2	2	-	STI	-	[12]
WSe2/MoS_2	p-WSe_2	−80 to 0	6	-	STI	-	[13]
$MoTe_2/SnS_2$	SnS_2	0 to 60	1	-	STI	-	[14]
MoS_2 /rubrene	p-Rubrene	−80 to 60	10	-	STI	-	[15]
BP/ReS_2	BP	−4 to 2	1	-	Non-volatile STI, Logic-in-memory	-	[16]
PTCDI-C13 /DNTT	PTCDI-C13	0 to 8	8	48 %	STI	-	[17]
PhC2-BQQDI /C8-BTBT	PhC2-BQQDI	0 to 9	12	-	STI	-	[18]
WSe_2/InSe	n-MOSFET	−100 to −70	1	-	Quaternary inverter	-	[19]
$WSe_2/MoTe_2$ /MoS_2	$WSe_2/MoTe_2$	−6 to −1	2	-	Quaternary inverter	-	[20]
DNTT /PTCDI-C13	PTCDI-C13	0 to 5	5	-	STI (Flash-memory)	-	[21]
$MoTe_2$	p-$MoTe_2$	−60 to 60	2	-	STI	-	[22]
$PdSe_2$ /Graphene /MoS_2	Resistor	−2 to 2	2	-	STI	-	[23]
C8-BTBT /PhC2-BQQDI	PhC2-BQQDI	0 to 6	10	-	Non-volatile STI, Logic-in-memory	-	[24]
**PTCDI-C13 /DNTT**	**PTCDI-C13**	**0 to 6**	**6**	**59 %**	**Non-volatile STI, Logic conversion in-memory**	**two-stage**	**This work**

STI, standard ternary inverter

Supplementary Table 2 | Comparison of static noise margin with the previously reported ternary logic devices.

Device type	Principle	Materials	Noise margin	Ref
T-CMOS	Band-to-band tunneling	Si	45 %	[25]
Embedded QDs	Quantized energy level	ZnO QDs	34 %	[26]
T-CMOS	Threshold switch	Si, NbO2	46.5 %	[27]
Heterojunction transistor	Zero transconductance	PTCDI-C8 / IGZO	-	[28]
**Heterojunction non-volatile memory**	**Negative transconductance**	**PTCDI-C13 / DNTT**	**59 %**	**This work**

T-CMOS, ternary CMOS; QDs, quantum dots;

Supplementary Table 3 | Comparison with the previously reported binary/ternary reconfigurable inverter.

Binary logic operation		Ternary logic operation		Integration level	Applications	Ref
V_{IN} / V_{OUT}	V_{DD}	V_{IN} / V_{OUT}	V_{DD}			
1 V / 0.2 V	0.2 V	1 V / 2 V	2 V	-	Binary/ternary reconfigurable inverter	[31]
30 V / 0.4 V	0.4 V	30 V / 2 V	2 V	-	Binary/ternary reconfigurable inverter	[32]
40 V / 0.1 V	0.1 V	40 V / 2 V	2 V	-	Binary/ternary reconfigurable inverter	[33]
60 V / 12 V	26 V	60 V / 26 V	26 V	-	Binary/ternary reconfigurable inverter	[34]
**6 V**				**Two-stage inverter**	**Binary/ternary reconfigurable inverter<br>STI / PTI / NTI<br>Logic conversion-in-memory**	**This work**

STI, standard ternary inverter; PTI, positive ternary inverter; NTI, negative ternary inverter;

References

1 Nourbakhsh, A., Zubair, A., Dresselhaus, M. S. & Palacios, T. s. Transport properties of a MoS2/WSe2 heterojunction transistor and its potential for application. *Nano Lett.* **16**, 1359-1366 (2016).

2 Huang, M. *et al.* Multifunctional high-performance van der Waals heterostructures. *Nat. Nanotechnol.* **12**, 1148-1154 (2017).

3 Shim, J. *et al.* Light-triggered ternary device and inverter based on heterojunction of van der waals materials. *ACS Nano* **11**, 6319-6327 (2017).

4 Kim, J. B. *et al.* Photosensitive graphene P–N junction transistors and ternary inverters. *ACS Appl. Mater. & Interfaces* **10**, 12897-12903 (2018).

5 Kobashi, K., Hayakawa, R., Chikyow, T. & Wakayama, Y. Multi-Valued Logic Circuits Based on Organic Anti-ambipolar Transistors. *Nano Lett.* **18**, 4355-4359 (2018).

6 Lv, W. *et al.* Multistate logic inverter based on black phosphorus/SnSeS heterostructure. *Adv. Electron. Mater.* **5**, 1800416 (2018).

7 Duong, N. T. *et al.* Modulating the functions of MoS2/MoTe2 van der Waals heterostructure via thickness variation. *ACS Nano* **13**, 4478-4485 (2019).

8 Hu, R., Wu, E., Xie, Y. & Liu, J. Multifunctional anti-ambipolar pn junction based on MoTe2/MoS2 heterostructure. *Appl. Phys. Lett.* **115**, 073104 (2019).

9 Wu, E. *et al.* Photoinduced doping to enable tunable and high-performance anti-ambipolar MoTe2/MoS2 heterotransistors. *ACS Nano* **13**, 5430-5438 (2019).

10 Yoo, H., On, S., Lee, S. B., Cho, K. & Kim, J. J. Negative Transconductance Heterojunction Organic Transistors and their Application to Full-Swing Ternary Circuits. *Adv. Mater.* **31**, 1808265 (2019).

11 Hassan, Y. *et al.* Phase-Engineered Molybdenum Telluride/Black Phosphorus Van der Waals Heterojunctions for Tunable Multivalued Logic. *ACS Appl. Mater. & Interfaces* **12**, 14119-14124 (2020).

12 Kim, B. Inkjet-printed ternary inverter circuits with tunable middle logic voltages. *Adv. Electron. Mater.* **6**, 2000426 (2020).

13 Kim, J. Y. *et al.* Distinctive field-effect transistors and ternary inverters using cross-type WSe2/MoS2 heterojunctions treated with polymer acid. *ACS Appl. Mater. & Interfaces* **12**, 36530-36539 (2020).

14 Kim, S. *et al.* Gate-switchable rectification in isotype van der Waals heterostructure of multilayer MoTe2/SnS2 with large band offsets. *npj 2D Materials and Applications* **4**, 15 (2020).

15 Park, C.-J. *et al.* Photo-responsive MoS2/organic-rubrene heterojunction field-effect-transistor: application to photo-triggered ternary inverter. *Semicond. Sci. Technol.* **35**, 065020 (2020).

16 Xiong, X. *et al.* Reconfigurable logic-in-memory and multilingual artificial synapses based on 2D heterostructures. *Adv. Funct. Mater.* **30**, 1909645 (2020).

17 Lee, C. *et al.* Systematic Control of Negative Transconductance in Organic Heterojunction Transistor for High-Performance, Low-Power Flexible Ternary Logic Circuits. *Small* **17**, 2103365 (2021).

18 Panigrahi, D., Hayakawa, R., Honma, K., Kanai, K. & Wakayama, Y. Organic heterojunction transistors for mechanically flexible multivalued logic circuits. *Applied Physics Express* **14**, 081004 (2021).

19 Paul Inbaraj, C. R. *et al.* A bi-anti-ambipolar field effect transistor. *ACS Nano* **15**, 8686-8693 (2021).

20 Son, H. *et al.* Complementary driving between 2D heterostructures and surface functionalization for surpassing binary logic devices. *ACS Appl. Mater. & Interfaces* **13**, 8692-8699 (2021).

21 Choi, J. *et al.* Vertically stacked, low-voltage organic ternary logic circuits including nonvolatile floating-gate memory transistors. *Nat. Commun.* **13**, 2305 (2022).

22 Geng, G. *et al.* Dielectric engineering enable to lateral anti-ambipolar MoTe 2 heterojunction. *Nanotechnology* **33**, 175704 (2022).

23 Lee, M. *et al.* Graphene Bridge Heterostructure Devices for Negative Differential Transconductance Circuit Applications. *Nano-Micro Letters* **15**, 1-11 (2023).

24 Panigrahi, D., Hayakawa, R., Zhong, X., Aimi, J. & Wakayama, Y. Optically Controllable Organic Logic-in-Memory: An Innovative Approach toward Ternary Data Processing and Storage. *Nano Lett.* (2023).

25 Jeong, J. W. et al. Tunnelling-based ternary metal–oxide–semiconductor technology. Nat. Electron. 2, 307-312 (2019).

26 Lee, L. et al. ZnO composite nanolayer with mobility edge quantization for multi-value logic transistors. Nat. Commun. 10, 1998 (2019).

27 Heo, S. et al. in 2021 IEEE International Electron Devices Meeting (IEDM). 32.32. 31-32.32. 34 (IEEE).

28 Lim, D. U., Jo, S. B., Kang, J. & Cho, J. H. Multi‐State Heterojunction Transistors Based on Field‐Effect Tunneling‐Transport Transitions. Adv. Mater. 33, 2101243 (2021).

29 Moon, H. *et al.* Synthesis of ultrathin polymer insulating layers by initiated chemical vapour deposition for low-power soft electronics. *Nat. Mater.* **14**, 628-635 (2015).

30 Yang, S. C. *et al.* Large-scale, low-power nonvolatile memory based on few-layer MoS2 and ultrathin polymer dielectrics. *Adv. Electron. Mater.* **5**, 1800688 (2019).

31 Huang, M. et al. Multifunctional high-performance van der Waals heterostructures. Nat. Nanotechnol. 12, 1148-1154 (2017).

32 Srivastava, P. K. et al. Multifunctional van der Waals broken‐gap heterojunction. Small 15, 1804885 (2019).

33 Wu, E. et al. Photoinduced doping to enable tunable and high-performance anti-ambipolar MoTe2/MoS2 heterotransistors. ACS Nano 13, 5430-5438 (2019).

34 Duong, N. T. et al. Modulating the functions of MoS2/MoTe2 van der Waals heterostructure via thickness variation. ACS Nano 13, 4478-4485 (2019).
